# Reduced Intra- and Extracellular Circulating Postprandial Lysosomal Acid Lipase Activity in Patients with MASLD

**DOI:** 10.3390/metabo14120725

**Published:** 2024-12-23

**Authors:** Monica Mischitelli, Eleonora Poggiogalle, Giulia Tozzi, Flaminia Ferri, Simona Parisse, Benedetta Meloni, Anna Morrone, Alice Sabbadini, Monther Salem, Elena Gangitano, Adriano De Santis, Giulia d’Amati, Lucio Gnessi, Lorenzo Maria Donini, Stefano Ginanni Corradini

**Affiliations:** 1 Department of Translational and Precision Medicine, Sapienza University of Rome, 00185 Rome, Italy; monica.mischitelli@uniroma1.it (M.M.); flaminia.ferri@uniroma1.it (F.F.); simona.parisse@uniroma1.it (S.P.); benedetta.meloni@policlinico.mi.it (B.M.); anna.morrone@asp.crotone.it (A.M.); alice.sabbadini@unicampus.it (A.S.); salem.1787450@studenti.uniroma1.it (M.S.); adriano.desantis@uniroma1.it (A.D.S.); 2 Department of Experimental Medicine, Sapienza University of Rome, 00185 Rome, Italy; eleonora.poggiogalle@uniroma1.it (E.P.); elena.gangitano@uniroma1.it (E.G.); lucio.gnessi@uniroma1.it (L.G.); lorenzomaria.donini@uniroma1.it (L.M.D.); 3 Division of Metabolic Diseases, Bambino Gesù Children’s Hospital IRCCS, 00146 Rome, Italy; giulia.tozzi@opbg.net; 4 Department of Medical-Surgical Sciences and Biotechnologies, Sapienza University of Rome, 00185 Rome, Italy; giulia.damati@uniroma1.it

**Keywords:** metabolic dysfunction, lysosomal acid lipase, PNPLA3 polymorphism, metabolic flexibility

## Abstract

Background/Objectives: Low fasting blood lysosomal acid lipase (LAL) activity is associated with the pathogenesis of metabolic hepatic steatosis. We measured LAL activity in blood and plasma before and after an oral fat tolerance test (OFTT) in patients with metabolic-dysfunction-associated steatotic liver disease (MASLD). Methods: Twenty-six controls and seventeen patients with MASLD but without diabetes were genotyped for the patatin-like phospholipase 3 (PNPLA3) rs738409 variant by RT-PCR and subjected to an OFTT, measuring LAL activity in blood and plasma with a fluorimetric method. Results: LAL activity in blood both under fasting and 4 h after OFTT (0.846 ± 0.309 nmol/spot/h vs. 1.180 ± 0.503 nmol/spot/h *p* < 0.01) was lower in patients with MASLD compared to controls. These differences were present only in carriers of the PNPLA3 variant. In controls not carrying the PNPLA3 variant, the postprandial increase in blood LAL activity was negatively correlated with that of serum triglycerides (*p* < 0.05). Extracellular LAL activity in plasma was lower in patients with MASLD (n = 9) compared to controls (n = 8) in the fasting state (*p* < 0.01) and 4 h post-meal (*p* < 0.05). The area under the curve up to 6 h of plasma LAL activity was lower in patients with MASLD than in controls (*p* < 0.05) and correlated negatively with that of triglycerides only in controls (r = −0.841; *p* < 0.01). Conclusions: Patients with MASLD have reduced LAL activity in blood and plasma both before and 4 h after a meal. In patients with MASLD, the physiological negative correlation between circulating LAL levels and postprandial hypertriglyceridemia is lost.

## 1. Introduction

Metabolic-dysfunction-associated steatotic liver disease (MASLD) has a very high prevalence and is characterized by the accumulation of lipid droplets (LDs) in hepatocytes consisting mainly of triglycerides and cholesterol [1,2]. The pathogenesis of non-alcoholic fatty liver disease (NAFLD), the previous definition of MASLD, has been associated with the suboptimal functioning of two closely interconnected mechanisms of cellular catabolism of LDs, lipolysis, and lipophagy [3,4]. Indeed, insufficient lipolysis due to the rs738409 genetic variant of patatin-like phospholipase 3 (PNPLA3) has been demonstrated in NAFLD patients [4]. Furthermore, NAFLD patients have an epigenetic reduction in fasting blood activity of the lipophagic enzyme, lysosomal acid lipase (LAL) [5,6,7]. This reduction in LAL enzymatic activity has been demonstrated by whole blood measurements that correlate with those in the liver and appears to relate to low enzymatic activity within platelets [7,8].

Patients with NAFLD/MASLD are at significantly increased risk of cardiovascular disease. This risk includes elevated fasting plasma triglyceride concentrations, large VLDL, elevated small dense LDL concentrations, low HDL-cholesterol (HDL-C) concentrations, and high triglyceride/HDL-C ratios [6,9,10,11,12,13]. Interestingly, a very similar fasting plasma lipid pattern is present in patients with severe genetic deficiency of LAL and intravenous administration of recombinant LAL is able to correct this dyslipidemia [14].

Another characteristic of patients with NAFLD is poor postprandial metabolic flexibility [15]. Indeed, a high plasma triglyceride/HDL-C ratio better discriminates patients with NAFLD compared to healthy controls when measured in postprandial conditions and predicts cardiovascular disease even in patients with low LDL-C treated with statins [13,16,17]. Consequently, there is great interest in trying to define the complex, largely unexplored pathogenetic mechanisms of the excessive hypertriglyceridemic response to fatty meals in both the fields of MASLD and cardiovascular disease. Among these mechanisms, in addition to intracellular lipolysis and lipophagy, extracellular lipolysis by intravascular lipases—such as hepatic lipase, endothelial lipase, and, above all, lipoprotein lipase (LPL)—plays an important role [18]. Although experimental studies have demonstrated that LAL can be secreted/exocytosed from cells, no measurements of extracellular plasma LAL activity have been reported in humans in vivo either under fasting conditions or after a meal [5,19,20,21,22,23].

In the present study, we aimed to compare the postprandial response to a standard fatty meal of LAL activity in whole blood in patients with MASLD and controls with healthy liver and correlate it with plasma lipid values. We then wanted to verify whether the carrier status of the PNPLA3 rs738409 variant was associated with LAL activity levels in blood, both in patients with MASLD and in controls. Finally, we wanted to investigate whether extracellular LAL activity was measurable in plasma, investigating its postprandial variations and correlations with plasma lipids in patients with MASLD and controls.

## 2. Materials and Methods

### 2.1. Study Population

We recruited 17 patients with MASLD and 26 controls with healthy livers [1]. Controls were recruited from laboratory staff, faculty, residents, and students. In all participants, liver ultrasonography was performed by the same operator, who was blinded to the clinical and laboratory data, and the presence or absence of hepatic steatosis was assessed ultrasonographically according to the criteria of Hamaguchi et al. [24]. Advanced fibrosis was excluded by liver biopsy in 9 out of 17 patients with MASLD and by Fibrosis-4 (FIB-4) score in the others [25,26].

The inclusion criteria for all participants were to be aged > 18 and <70 years and be Italian of Caucasian ethnicity. The exclusion criteria (see Supplementary Methods) concerned diseases, including diabetes, and therapies, including statins, that may influence lipid metabolism, with the exception of non-syndromic overweight/obesity with BMI < 35 kg/m^2^ and mild dyslipidemia.

### 2.2. Oral Fat Tolerance Test

A standard high-fat meal was consumed as an oral fat tolerance test (OFTT) in the morning as breakfast, after a 12-h fast (see Supplementary Methods and Table S1). For all subjects, whole blood LAL activity was measured at fasting baseline (T0) and 4 h after ingestion of the fatty meal (T4). In the last 17 participants (9 patients with MASLD and 8 controls) in whom LAL extracellular activity in plasma was also measured, postprandial blood samples were taken every 2 h until the sixth hour after the end of the meal (T2, T4, T6).

### 2.3. Analytical Methods

LAL enzymatic activity in whole blood was measured using 75 µL of blood in ethylene-diamine-tetra acetic acid (EDTA), with the dry blood spot (DBS) technique described by Hamilton et al. [7,27] (Supplementary Methods). Extracellular LAL activity in plasma was measured from blood in sodium citrate. Immediately after collection, 3 mL of blood was centrifuged at 1500× *g* at 4 °C for 10 min. Then, 200 µL of supernatant plasma was immediately carefully collected without disturbing the white buffy layer, frozen at −20 °C, and analyzed within 1 week, as described by Hamilton et al. for LAL activity analysis from DBS samples [27].

Plasma LPL enzymatic activity was measured with a fluorometric assay (Cell biolabs. Inc 7758 San Diego, CA, USA) (Supplementary Methods).

Measurements of triglycerides, LDL-C, HDL-C, serum aminotransferases, and white blood cell and platelet counts were performed using standard methods (Supplementary Methods).

### 2.4. Polymorphism Screening

To assess the rs738409 C > G (I148M PNPLA3), EDTA blood, obtained by venepuncture, was collected. Assay was carried out in duplicate by TaqMan^®^ SNP Genotyping Assays (Life Technologies, Carlsbad, CA, USA) according to the protocols. PCR allelic discrimination was performed twice in two independent analyses in order to obtain a 100% concordance rate.

### 2.5. Statistical Analysis

An a priori estimate of the sample size was not possible given the lack of postprandial LAL activity data. In a previous study, we measured LAL activity in fasting whole blood in patients with NAFLD and controls without any liver disease whose average was, respectively, 1.15 nmol/spot/h and 0.60 nmol/spot/h [6]. Using these averages and a pooled standard deviation of 0.56 nmol/spot/h, we calculated that it would be necessary to recruit a minimum of 17 participants per group to detect differences with 80% power and alpha less than 0.05. We enrolled 10 additional controls to quantify any physiological correlations between LAL activity in blood and serum lipids.

Data were expressed as mean ± SD. Fisher’s exact test or chi-square test was used to compare categorical variables. For continuous variables, the presence or absence of normal distribution was assessed using the Shapiro–Wilk test. For measurements taken in the fasting state and 2, 4, and 6 h after OFTT in the two groups—patients with MASLD and controls—differences were assessed using repeated measures ANOVA with within-subjects tests, i.e., the mean of the change for the average individual case regardless of group membership, and between-subjects tests, i.e., comparing the temporal changes in the two groups. Data were log10 transformed to obtain a normal distribution before performing the repeated measures ANOVA. Differences between patients with MASLD and controls at the same time point and those at each time point from baseline were assessed using Student’s *t* test for independent samples and paired samples, respectively. For measurements made in the fasting state and only 4 h after OFTT, differences between patients with MASLD and controls at each time point were assessed using the Mann–Whitney U test or independent samples *t* test, as appropriate. Differences in T4 values from baseline within each of the two groups were performed using Wilcoxon’s test or paired *t*-test, as appropriate.

Since *p*-values can be influenced by population size, if the *p*-value was significant, the results of comparisons were also reported as effect sizes (partial eta-squared or Cohen’s d or r values) [28,29]. The interpretation of effect sizes was chosen as reported by Cohen and based on the respective probability of superiority value [28,29]. The probability of superiority is “the percentage of occasions on which a randomly selected member of the distribution with the higher mean will have a higher score than a randomly selected member of the other distribution”. [29]. For the within-subject and between-subject tests of the repeated-measures ANOVA, a partial eta-squared value > 0.010 < 0.059 was considered a small effect, 0.06 < 0.14 a medium effect, and ≥0.14 a large effect. In the case of simple effects tests to compare two groups at each time point or two time points within the same group, we reported Cohen’s d values expressing the point estimate for parametric tests. We calculated effect size r values for nonparametric tests with the formula r = Z/√N, where Z is the value of the standardized test statistic Z reported in the SPSS output and N is the number of observations. We considered Cohen’s d values > 0.2 < 0.5 and r values > 0.10 < 0.24 as a weak effect, Cohen’s d values 0.50 < 0.80 and r values 0.24 < 0.37 as a moderate effect, and Cohen’s d values ≥ 0.80 and r ≥ 0.37 as a strong effect [29]. The area under the curve (AUC) of parameters measured during the test meal was calculated using the trapezoid method. Pearson’s test was used to evaluate correlations between lipase activities and serum lipids. Statistical analyses were performed using the Statistical Program for Science (SPSS) version 28 for Windows and Prism GraphPad version 10. Differences were considered statistically significant at *p* < 0.05.

## 3. Results

We studied 17 patients with MASLD and 26 controls without any liver disease with OFTT. Liver biopsy was performed in 9 out of 17 patients with MASLD. In these patients, fibrosis was absent in one case and the fibrosis score was 1 in the other cases. All patients with MASLD who did not undergo liver biopsy had a FIB-4 < 1.3 and were, therefore, considered at low risk of advanced fibrosis. The demographic, metabolic, and clinical data are reported in Table 1. Patients with MASLD, compared to controls, were significantly older and had higher values of BMI, serum AST and ALT, and plasma LDL-C and triglycerides, while they had lower plasma HDL-C values. No subjects enrolled in the study had diabetes or arterial hypertension. In the MASLD group, the prevalence of obesity, overweight, and dyslipidemia was 47%, 53%, and 65%, respectively. Only 23% of the controls were overweight, with no other metabolic alterations. Although platelet and leukocyte counts did not differ between the two groups, fasting whole blood LAL activity was significantly lower in patients with MASLD compared to controls.

### 3.1. Fasting and 4-h Postprandial Plasma Triglyceride Concentrations in Patients with MASLD and Healthy Liver Controls

**Figure 1:**
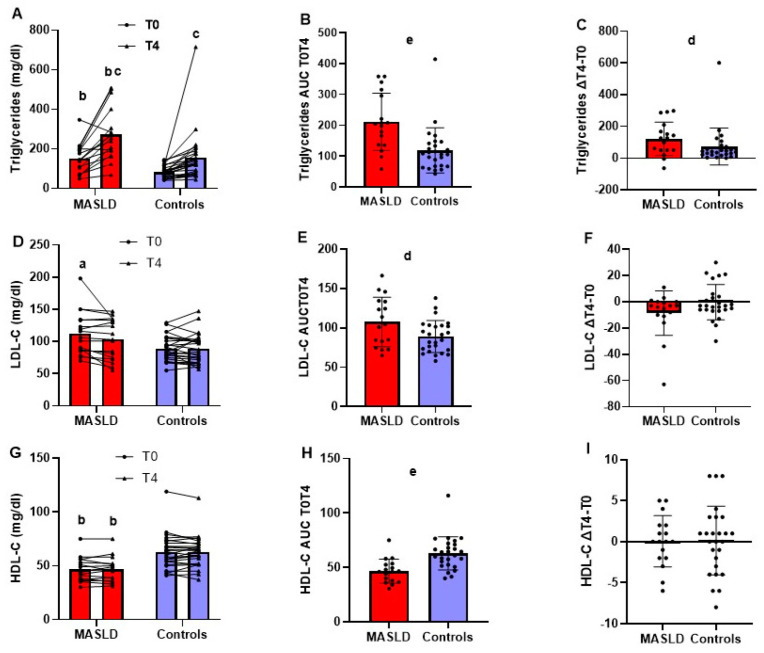
Plasma lipids before and after 4 h from the oral fat tolerance test. Fasting and 4-h post-OFTT plasma lipids in 17 patients with MASLD and 26 controls. Concentrations at T0 and T4 (triglycerides, panel (**A**); LDL-C, panel (**D**); HDL-C, panel (**G**)), reported as dot plots and lines connecting the same patient and means; AUC T0T4 of the concentrations of each plasma lipid (triglycerides, panel (**B**); LDL-C, panel (**E**); HDL-C, panel (**H**)) and the difference in the concentrations of each lipid at T4 minus that at T0 (∆T4 − T0) (triglycerides, panel (**C**); LDL-C, panel (**F**); HDL-C, panel (**I**)), reported as dot plots and means ± SD. ^a^ *p* < 0.05, ^b^ *p* < 0.01 patients with MASLD vs. controls at the same time point; ^c^ *p* < 0.01 T4 vs. T0 within the same group; ^d^ *p* < 0.01, ^e^ *p* < 0.001 patients with MASLD vs. controls. (Student’s *t* test for independent or paired samples or Mann–Whitney U test or Wilcoxon’s test as appropriate.) AUC, area under the curve; ΔT4 − T0 difference between the value measured at T4 and that at T0; LDL-C = LDL cholesterol, MASLD, metabolic-dysfunction-associated steatotic liver disease; HDL-C = HDL cholesterol.

Figure 1, panel A, shows the individual data and mean plasma triglyceride concentrations in 17 patients with MASLD and 26 controls before and after 4 h of OFTT. Patients with MASLD compared to controls had higher values, with a strong effect size of triglyceride concentration at T0 (*p* < 0.001; Cohen’s d = 1.322) and T4 (*p* < 0.001; effect size r = 0.528). Furthermore, triglycerides were significantly higher at T4 than at T0 in both patients with MASLD (*p* < 0.001; Cohen’s d = 1.15) and controls (*p* < 0.001; effect size r = 0.680), with a strong effect size. Patients with MASLD, compared with controls, had a higher AUC of triglycerides T0T4 (*p* < 0.001; effect size r = 0.527) (Figure 1, panel B) and higher values—with a moderate effect size—of the difference between triglyceride concentrations at T4 and T0 (*p* = 0.028; effect size r = 0.335) (Figure 1, panel C).

### 3.2. Fasting and 4-h Postprandial Plasma LDL-C and HDL-C Concentrations in Patients with MASLD and Healthy Liver Controls

Figure 1 panels D and G show the individual data and mean plasma LDL-C and HDL-C concentrations, respectively, in the 17 patients with MASLD and 26 controls before and after 4 h of OFTT. Patients with MASLD compared to controls had higher LDL-C concentrations with a strong effect size at T0 (*p* = 0.018; Cohen’s d = 0.881) and lower HDL-C concentrations with a strong effect size at T0 (*p* < 0.001; effect size r = 0.535) and T4 (*p* < 0.001; effect size r = 0.561). Both LDL-C and HDL-C did not differ at T4 and T0 in both groups. Patients with MASLD compared with controls had a higher LDL-C AUC T0–T4 with a moderate effect size (*p* = 0.039; Cohen’s d = 0.741) (Figure 1, panel E) and HDL-C AUC T0T4 with a strong effect size (*p* < 0.001; effect size r = 0.553) (Figure 1, panel H). There were no differences between groups regarding the difference between the T4 and T0 concentrations of LDL-C (Figure 1, panel F) and HDL-C (Figure 1, panel I).

### 3.3. Fasting and 4-h Postprandial Plasma Triglyceride/HDL-C Ratio in Patients with MASLD and Healthy Liver Controls

Patients with MASLD compared with controls had higher plasma triglyceride/HDL-C ratio values, with a strong effect size at T0 (*p* < 0.001; effect size r = 0.583) and T4 (*p* < 0.001; effect size r = 0.591) (Supplementary Figure S1, panel A). Furthermore, the triglyceride/HDL-C ratio was significantly higher at T4 than at T0 in both patients with MASLD (*p* = 0.002; effect size r = 0.480) and controls (*p* < 0.001; effect size r = 0.680), with a strong effect size (Supplementary Figure S1, panel A). Patients with MASLD compared with controls had higher values, with a strong effect size of AUC T0T4 (*p* < 0.001; effect size r = 0.606) (Supplementary Figure S1, panel B) and higher values, with moderate effect size, of the difference between the triglyceride/HDL-C ratio at T4 and T0 (*p* = 0.016; effect size r = 0.367) (Supplementary Figure S1, panel C).

### 3.4. Fasting and 4-h Postprandial Whole Blood LAL Activity in Patients with MASLD and Healthy Liver Controls

**Figure 2:**
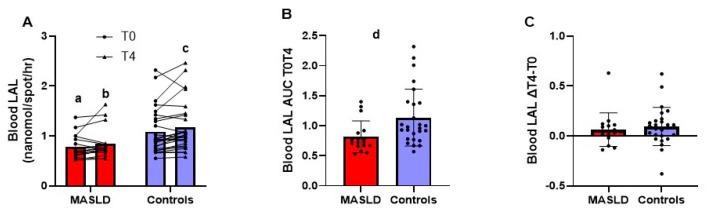
LAL enzymatic activity in whole blood before and after 4 h from the oral fat tolerance test in patients with MASLD and controls. Panel (**A**) shows measurements at T0 and T4 reported as dot plots and lines. In panel (**B**) is shown the AUC T0T4 of enzyme activity and finally, in panel (**C**) is shown the difference in enzyme activity T4 minus T0 (∆T4 − T0) reported as dot plots and means ± SD. ^a^ *p* < 0.05, ^b^ *p* < 0.01 patients with MASLD vs. controls at the same time point (Mann–Whitney U test); ^c^ *p* < 0.01 T4 vs. T0 in controls (Wilcoxon’s test); ^d^ *p* < 0.01 patients with MASLD vs. controls (Mann–Whitney U test). AUC, area under the curve; ΔT4 − T0 difference between the value measured at T4 and T0; LAL, lysosomal acid lipase; MASLD, metabolic-dysfunction-associated steatotic liver disease.

Figure 2, panel A, shows the individual data and mean LAL enzymatic activity in whole blood before and after 4 h from the OFTT. Blood LAL activity was significantly lower in patients with MASLD compared to controls with a strong effect size, both at T0 (0.784 ± 0.239 nmol/spot/h vs. 1.086 ± 0.464 nmol/spot/h; *p* = 0.014; effect size r = 0.375) and at T4 (0.846 ± 0.309 nmol/spot/h vs. 1.180 ± 0.503 nmol/spot/h; *p* = 0.004; effect size r = 0.441). Of note, LAL activity in whole blood was significantly higher at T4 compared to T0 only in controls with a strong effect size (*p* = 0.008; effect size r = 0.406), while it did not significantly change in the MASLD group (*p* = 0.089). The AUC T0T4 of blood LAL activity of the MASLD group was significantly lower (*p* = 0.006; effect size r = 0.421) than that of the controls with a strong effect size (Figure 2, panel B). However, as shown in Figure 2, panel C, the difference between LAL activity at T4 and T0 was not significantly different between the two groups (*p* = 0.345).

Figure 2 displays the blood LAL activity at fasting and 4 h after OFTT in 17 patients with MASLD and 26 controls. Measurements at T0 and T4 are reported as dot plots and the lines connect the same patient and means (Figure 2A). Additionally, the AUC T0T4 of enzyme activity (Figure 2B) and difference in enzyme activity T4 minus T0 (∆T4 − T0) (Figure 2C) are reported as dot plots and means ± SD.

Concerning the relationship between fasting blood LAL activity or its postprandial changes and plasma lipids, we found no correlation between blood LAL activity at T0 or T4 and any of the plasma lipids at the same time points (Supplementary Table S2) in either group. Table 2 shows Pearson’s r correlation coefficients and the corresponding *p* values between the difference in blood LAL activity at T4 minus T0 and lipid parameters. In controls, the difference in blood LAL activity at T4 minus T0 was negatively correlated with LDL-C concentrations at T0 and T4 but not with the difference in LDL-C concentrations at T4 minus T0. Furthermore, in controls, the difference in blood LAL activity at T4 minus T0—although not correlated with triglyceride concentration and the triglyceride/HDL-C ratio at T0—was strongly negatively correlated with triglyceride concentration and the triglyceride/HDL-C ratio at T4 and with the difference in these two parameters at T4 minus T0. In patients with MASLD, no correlation was found between the difference in blood LAL activity at T4 minus that at T0 and any lipid parameter.

### 3.5. Fasting and 4-h Post-Meal LAL Activity in Whole Blood Depending on the Absence or Presence of the PNPLA3 rs738409 Variant in Patients with MASLD and Healthy Liver Controls

In the group of 26 controls, the rs738409 PNPLA3 variant was present in heterozygosity in 13 subjects and absent in the other 13 subjects. In the group of 17 patients with MASLD, the PNPLA3 variant was present in homozygosity in 4 patients, in heterozygosity in 6 subjects, and absent in 6 patients. In one patient with MASLD, this was indeterminable. We, therefore, wanted to verify whether the fasting and 4-h post-meal LAL blood activity behaved differently depending on the absence or presence of the PNPLA3 mutation. For this reason, we compared the 6 wild-type patients with MASLD (MASLD-Wt) with the 13 wild-type controls (Wt-controls) (Supplementary Table S3) and, separately, the 10 patients with MASLD with at least one mutated allele of the variant (MASLD-I148M) with the 13 controls with one mutated allele of the variant (-I148M controls) (Supplementary Table S4). Patients with MASLD were significantly older and had higher BMI values compared to controls, both in wild-type subjects and in those carrying the PNPLA3 variant; however, plasma lipids and fasting blood LAL activity differed significantly between patients with MASLD and controls only in the presence of the PNPLA3 variant. The prevalence of dyslipidemia and obesity in patients with MASLD carrying the PNPLA3 variant was 60% and 30%, respectively. The prevalence of dyslipidemia and obesity in patients with MASLD wild-type for PNPLA3 was 67% for both metabolic variables.

**Figure 3:**
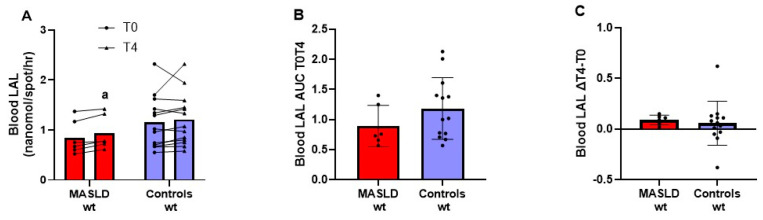
LAL enzymatic activity in whole blood before and after 4 h of oral fat tolerance test in patients with MASLD and controls without the rs738409 PNPLA3 variant. LAL activity in blood at fasting and 4 h after OFTT in 6 patients with MASLD and 13 controls who were wild type for the rs738409 PNPLA3 variant. Measurements at T0 and T4 reported as dot plots and lines connecting the same patient and means (**A**); AUC T0T4 of enzyme activity (**B**) and difference in enzyme activity T4 minus T0 (∆T4 − T0) (**C**) reported as dot plots and means ± SD. ^a^ *p* < 0.01. T4 vs. T0 in patients with MASLD (Student’s *t* test for paired samples). Abbreviations: AUC, area under the curve; ΔT4 − T0 difference between the value measured at T4 and T0; LAL, lysosomal acid lipase; MASLD, metabolic-dysfunction-associated steatotic liver disease; Wt, rs738409 PNPLA3 wild type.

Figure 3, panel A, shows the individual data and mean LAL enzyme activity in whole blood before and after 4 h of OFTT in PNPLA3 wild-type subjects. LAL activity in blood did not differ between patients with MASLD and controls, either at T0 (*p* = 0.21) or at T4 (*p* = 0.262). LAL activity in whole blood was significantly higher at T4 than at T0 only in patients with MASLD with a strong effect size (*p* = 0.006; Cohen’s d = 1.885), while it did not change significantly in controls (*p* = 0.359). In subjects without the PNPLA3 mutation, there were no significant differences in both AUC T0T4 (*p* = 0.226) (Figure 3, panel B) and the difference between LAL activity at T4 and T0 (*p* = 0.356) (Figure 3, panel C).

**Figure 4:**
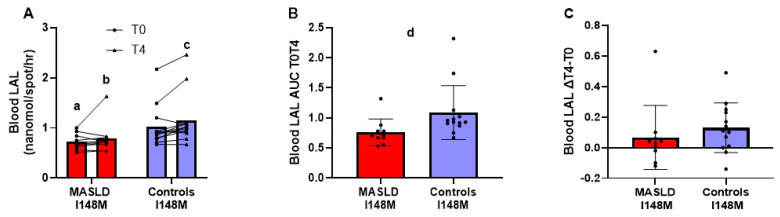
LAL enzyme activity in whole blood before and after 4 h of oral fat tolerance test in patients with MASLD and controls carrying the rs738409 PNPLA3 variant. LAL activity in blood at fasting and 4 h after OFTT in 6 patients with MASLD and 13 controls who were carriers of the rs738409 PNPLA3 variant. Measurements at T0 and T4 reported as dot plots and lines connecting the same patient and means (**A**); AUC T0T4 of enzyme activity (**B**) and difference in enzyme activity T4 minus T0 (∆T4 − T0) (**C**) reported as dot plots and means ± SD. ^a^ *p* < 0.05, ^b^ *p* < 0.01 patients with MASLD vs. controls at the same time point (Mann–Whitney U test). ^c^ *p* < 0.01 T4 vs. T0 in controls (Wilcoxon’s test). ^d^ *p* < 0.01 patients with MASLD vs. controls (Mann–Whitney U test). Abbreviations: AUC, area under the curve; ΔT4 − T0 difference between the value measured at T4 and T0; I148M, PNPLA3 rs738409 variant carriers; LAL, lysosomal acid lipase; MASLD, metabolic-dysfunction-associated steatotic liver disease.

In subjects carrying the PNPLA3 mutation (Figure 4, panel A), LAL activity in blood was significantly lower in patients with MASLD compared to controls, with strong effect size both at T0 (*p* = 0.018; effect size r = 0.492) and at T4 (*p* = 0.005, effect size r = 0.589). In subjects with the PNPLA3 mutation, LAL activity in whole blood was significantly higher at T4 compared to T0 only in controls, with a strong effect size (*p* = 0.017; effect size r = 0.499), while it did not change significantly in patients with MASLD (*p* = 0.358). As shown in Figure 4, panel B, in subjects with the PNPLA3 mutation, the AUC T0T4 of blood LAL activity in the MASLD group was significantly lower than that in the controls, with a strong effect size (*p* = 0.004; effect size r = 0.633). However, the difference between blood LAL activity at T4 and T0 was not significantly different between the two groups (*p* = 0.166) (Figure 4, panel C).

In the controls-Wt group, but not in the controls-I148M group, we found correlations between the difference in blood LAL activity at T4 minus that at T0 and lipid parameters, similar to those that we found when analyzing the entire cohort of controls. In fact, in the controls-Wt group, the difference in LAL activity in the blood at T4 minus that at T0 was negatively correlated with the values of the LDL-C at T0 but not with the difference in this parameter between T4 and T0 (Supplementary Table S5). Furthermore, in the controls-Wt group, the difference in blood LAL activity at T4 minus that at T0—although not correlated with the triglycerides concentration at T0—was negatively correlated with the difference in this parameter between T4 and T0.

### 3.6. Plasma Lipid Levels and Plasma Extracellular LAL Activity in the Fasting and Post-Meal State for up to 6 H in Patients with MASLD and Healthy Liver Controls

In a subgroup of MASLD (n = 9) and control (n = 8) patients (Supplementary Table S6), we wanted to measure the LAL extracellular activity in plasma, verifying its time course up to six hours after the meal and comparing it with the LPL activity.

Regarding the AUC T0-T6 of plasma lipids, patients with MASLD compared to controls showed significantly lower values of HDL-C and higher values of triglycerides and the triglyceride/HDL-C ratio (Table 3).

**Figure 5:**
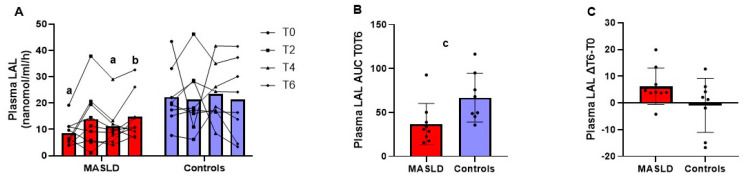
LAL enzyme activity in plasma at fasting and 2, 4, and 6 h after OFTT in 9 patients with MASLD and 8 controls. Measurements at each time point reported as dot plots and lines connecting the same patient and means (**A**); AUC T0T6 of enzyme activity (**B**) and difference in enzyme activity T6 minus T0 (∆T4 − T0) (**C**) reported as dot plots and means ± SD. ^a^ *p* < 0.01 patients with MASLD vs. controls at the same time point (Student’s *t* test for independent samples). ^b^ *p* < 0.05 T4 vs. T0 in patients with MASLD (Student’s *t* test for paired samples). ^c^ *p* < 0.05 patients with MASLD vs. controls. Abbreviations: AUC, area under the curve; LAL, lysosomal acid lipase; MASLD, metabolic-dysfunction-associated steatotic liver disease.

Figure 5, panel A, shows the individual data and mean plasma LAL activity at baseline and 2, 4, and 6 h after the test meal. The repeated measures ANOVA was not significant for the within-subject main effects test over time (*p* = 0.099), i.e., the mean of the change for the average individual case regardless of group membership. However, the between-subjects effect test, i.e., comparing the temporal changes in the two groups, was significant with a large effect size (*p* = 0.018; partial eta-squared = 0.318). Plasma LAL activity was significantly lower in patients with MASLD compared to controls, both at T0 (*p* = 0.002; Cohens d = 1.859) and T4 (*p* = 0.007, Cohens d = 1.502) with strong effect size, while it did not differ at T2 (*p* = 0.151) and T6 (*p* = 0.585). In patients with MASLD, postprandial plasma LAL activity compared to baseline was significantly higher only at T6 (*p* = 0.014; Cohen’s d = 1.042) with strong effect size, while there were no differences at other time points. In controls, no difference was present between postprandial plasma LAL activity compared to the baseline. The mean AUC T0T6 of LAL activity in the plasma of the MASLD group was significantly lower (*p* = 0.016; effect size r = 0.584) than that of the controls with a strong effect size (Figure 5, panel B). However, the difference between LAL activity at T6 and T0 was not significantly (*p* = 0.108) different between the two groups (Figure 5, panel C).

**Figure 6:**
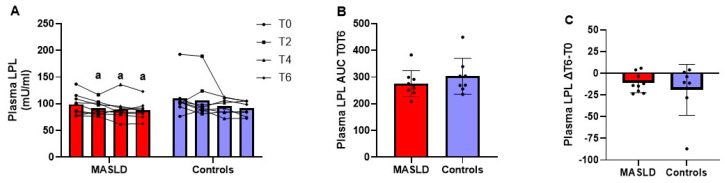
LPL enzyme activity in plasma before and up to 6 h after oral fat tolerance test in patients with MASLD and controls. LPL activity in plasma at fasting and 2, 4, and 6 h after OFTT in 9 patients with MASLD and 8 controls. Measurements at each time point reported as dot plots and lines connecting the same patient and means (**A**); AUC T0T6 of enzyme activity (**B**) and dif-ference of enzyme activity T6 minus T0 (∆T4 − T0) (**C**) reported as dot plots and means ± SD. ^a^ *p* < 0.05 each time point vs. T0 in patients with MASLD (Student’s *t* test for paired samples). AUC, area under the curve; LPL, Lipoprotein lipase; MASLD, metabolic dysfunction-associated steatotic liver disease.

Figure 6, panel A, shows the individual data and mean plasma LPL activity at baseline and 2, 4, and 6 h after the test meal. The repeated measures ANOVA was not significant for either the within-subject main effects test over time (*p* = 0.777) or the between-subjects effect test (*p* = 0.360).

No difference was found between patients with MASLD and controls in plasma LPL activity at any time point. In patients with MASLD, LPL activity at T2 (*p* = 0.042; Cohen’s d 0.808), T4 (*p* = 0.035; Cohen’s d 0.847), and T6 (*p* = 0.019; Cohen’s d 0.979) was significantly lower than at T0, with strong effect sizes. In controls, there were no significant differences at any of the post-meal time points compared to baseline. There were no differences between patients with MASLD and controls in LPL T0T6 AUCs (Figure 6, panel B) and the difference in enzyme activity at T6 minus that at T0 (Figure 6, panel C).

**Figure 7:**
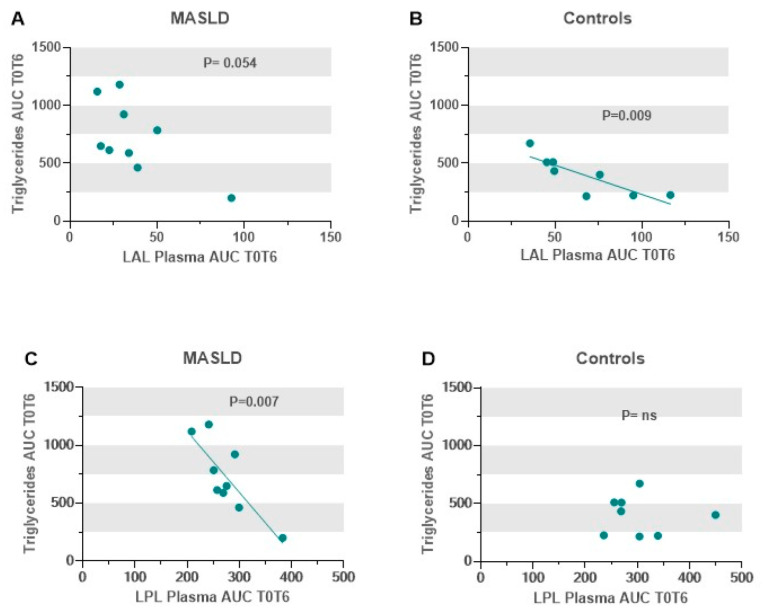
Scatter plots between the AUC of the plasma triglyceride concentration and those of the plasma enzymatic activities of LAL and LPL during a 6-h oral fat tolerance test. Triglycerides vs. LAL in patients with MASLD (**A**) and in controls (**B**). Triglycerides vs. LPL in patients with MASLD (**C**) and in controls (**D**) (Pearson correlations). Abbreviations: AUC, area under the curve; LAL, lysosomal acid lipase; LPL, lipoprotein lipase; MASLD, metabolic-dysfunction-associated steatotic liver disease.

As shown in Figure 7, in controls, the AUC T0T6 of plasma extracellular LAL activity negatively correlated with the AUC T0T6 of triglycerides (r = −0.841; *p* = 0.009), whereas the latter did not correlate with the AUC T0T6 of plasma LPL (r = −0.055; *p* = 0.897). On the contrary, in MASLD, the AUC T0T6 of plasma LPL activity correlated negatively with the AUC T0T6 of triglycerides (r = −0.820; *p* = 0.007), while the correlation between the latter and the AUC T0T6 of plasma LAL did not reach statistical significance (r = −0.658; *p* = 0.054).

Consequently, in controls, the AUC T0T6 of the triglyceride/HDL-C ratio correlated negatively only with the AUC T0T6 of the LAL plasma activity (r = −0.803; *p* = 0.016) and, on the contrary, in patients with MASLD, the AUC T0T6 of the triglyceride/HDL-C ratio correlated negatively only with that of LPL (r = −0.739; *p* = 0.023) (Supplementary Figure S2).

The difference in extracellular LAL activity in plasma T4 minus T0 was negatively correlated with serum AST (r = −0.686; *p* = 0.041) and ALT (r = −0.707; *p* = 0.033) activity in patients with MASLD, while the temporal variation in plasma LAL activity was not correlated with either AST (r = −0.599; *p* = 0.116) or ALT (r = −0.642; *p* = 0.086) in controls (Supplementary Figure S3).

## 4. Discussion

Several studies have reported that patients with NAFLD have significantly lower fasting whole blood LAL activity compared to controls [5,6,7]. In this study, we confirmed this finding in non-diabetic patients with MASLD. Our most important result is that the difference in blood LAL activity between patients with MASLD and controls was even greater four hours after a standard high-fat meal. It is possible that this could be explained by the same molecular mechanism demonstrated by others in hepatocytes in vitro and in the livers of NAFLD patients under fasting conditions [8,30]. Indeed, Gomaraschi et al. demonstrated in HepG2 cells that intracellular accumulation of free fatty acids and, consequently, triglycerides, reduces LAL activity [8]. Furthermore, Carotti et al. demonstrated in hepatocytes a high rate of dysfunctional LAL enzyme in response to intracellular fat overload [30]. The dysfunctional enzyme accumulates in the cytoplasm without entering lysosomes and is caused by excessive ubiquitination of the protein [30]. We speculate that these mechanisms could also be present in blood cells both under fasting and post-meal conditions. We found that whole blood LAL activity increased 4 h postprandially compared to baseline within the control group with high statistical significance. From our data, we cannot draw firm conclusions as there was a lack of postprandial response of blood LAL activity in patients with MASLD. In fact, within the MASLD group, there was a trend for a postprandial increase in blood LAL activity that did not reach statistical significance. We cannot exclude that the smaller sample size of the MASLD group contributed to the lack of significance in the postprandial increase in activity. Indeed, the difference between blood LAL activity measured 4 h postprandially and baseline was not significantly different between the two groups. In controls, but not in patients with MASLD, we found negative correlations between the difference between postprandial and fasting values of blood LAL activity and plasma lipid variables. These correlations, although indirect, allow for some speculation about a possible metabolic protective role of postprandial increases in blood LAL activity. A negative correlation was previously reported, in fasting conditions, between plasma LDL-C concentration and LAL activity in the blood within its normal range [31]. In our study, we extend this observation by describing a negative correlation in controls between the postprandial increase in LAL activity in blood and fasting and postprandial LDL-C values in controls. These correlations, in the absence of correlations between postprandial changes in LDL-C and those of LAL activity, would indirectly suggest a possible beneficial metabolic effect of postprandial LAL only in the long-term in the liver but not immediately at the blood level. Several pieces of previously published data indirectly support our hypothesis. In fact, LAL activity in whole blood is positively correlated with that in the liver and hepatocytes could endocytose LAL derived from blood cells, a capacity already demonstrated for other cell types [19,20,32,33,34].

In our study, increased postprandial blood LAL activity in controls was not related to fasting triglycerides but was negatively correlated with increased postprandial triglyceride concentration. This allows us to speculate that the postprandial increase in LAL activity could have a role at the blood level in maintaining postprandial hypertriglyceridemia within certain limits.

Given the relevance of our new data regarding reduced blood LAL activity after a fatty meal in patients with MASLD, as a second aim of our study, we wanted to verify whether this was associated with the PNPLA3 polymorphism [4]. We found that, when considering wild-type subjects for PNPLA3, blood LAL activity did not differ between patients with MASLD and controls both at baseline and after the meal. On the contrary, in the presence of the PNPLA3 variant, the blood LAL activity of patients with MASLD was higher than that of controls both at baseline and after the meal. Furthermore, the inverse correlations between postprandial increase in blood LAL and plasma lipids that we had found in controls were present only in wild-type subjects but not in carriers of the PNPLA3 variant. Our study does not allow us to give a clear mechanistic explanation of why blood LAL activity is low in patients with MASLD compared to controls only in the presence of the PNPLA3 variant. Assuming that LAL redistributes between different cellular compartments, these data allow us to speculate that suboptimal intrahepatocyte lipolysis due to the PNPLA3 variant could, by accumulating more LDs, favor LAL ubiquitination and dysfunctionality with a lower redistribution of the functional enzyme between cells and a reduced metabolic flexibility [8,19,20,30,32,33].

The results of the third aim of our study strengthen our hypothesis that circulatory LAL activity is involved in the metabolic flexibility of triglycerides at the peripheral level. Indeed, we demonstrated the presence of extracellular LAL activity in plasma in vivo. Plasma LAL activity both at baseline and four hours after the meal, as in the case of LAL activity in whole blood, was significantly lower in patients with MASLD than in controls. Furthermore, in these experiments conducted up to 6 h after the meal, the AUC of plasma triglycerides was negatively correlated with that of extracellular plasma LAL activity only in controls, while it was negatively correlated with the AUC of plasma LPL only in patients with MASLD. These data, although not constituting direct evidence, allow us to speculate that patients with MASLD could have an impairment of the physiological postprandial intravascular LAL hydrolase activity for triglycerides, which is already hypothesized by others [33]. Indeed, mice with systemic genetic loss of LAL show increased triglyceride levels two hours after a fatty meal but not in the fasting state, consistent with plasma LAL triglyceride hydrolase activity [19]. However, our study was not designed to clarify from which cells plasma LAL derives nor whether it acts as an intravascular triglyceride hydrolase with a mechanism similar to “digestive esophagy” in the nucleus of aggregated LDL or by forming complexes with VLDL after secretion into extracellular vesicles or exosomes [23,35]. Finally, it is interesting to note that in patients with MASLD in our current study, we found a negative correlation between serum aminotransferases and increased plasma LAL activity four hours after the meal. This observation is supported by the association already demonstrated in NAFLD patients between low fasting blood LAL activity values and elevated serum ALT values [36]. Further studies are needed to clarify the relationships between blood LAL and hepatocyte damage.

A limitation of our study is that the control group was highly unbalanced compared to the MASLD group with respect to age and BMI. Therefore, the results should be verified in a larger controlled study that also includes a group matched for age and BMI. Furthermore, the small number of subjects enrolled, especially when subgrouping according to the PNPLA3 polymorphism or in the 6-h experiments, could have biased some analyses such as correlations and made it impossible to perform multivariate analyses.

## 5. Conclusions

In conclusion, in the present study, we demonstrated that in patients with MASLD, LAL activity in both blood and plasma is reduced before and 4 h after a meal. Furthermore, circulating LAL levels are negatively correlated with postprandial triglyceride increases only in controls and not in patients with MASLD. In the latter, postprandial hypertriglyceridemia is negatively correlated with LPL activity. These data, although indirect, allow us to speculate about reduced metabolic flexibility in patients with MASLD based on reduced circulating LAL activity. Larger studies are needed to clarify the relationships between reduced circulating LAL activity, the presence of the PNPLA3 variant, MASLD, and hepatocyte damage. Finally, the origin, function, and regulation of extracellular circulating LAL activity should be better characterized in the future.

## Figures and Tables

**Table 1:** Demographic, clinical, and fasting metabolic characteristics of patients with MASLD and the healthy liver control group.

	MASLD (n = 17)	Controls (n = 26)	*p*
Men, n (%)	9 (52.9)	9 (34.6)	0.234
Age, y	48.24 ± 12.38	25.42 ± 3.50	<0.001
Body Mass Index, kg/m^2^	30.4 ± 3.2	22.6 ± 2.9	<0.001
Obesity, n (%)	8 (47.1)	0 (0)	<0.001
Overweight, n (%)	9 (52.9)	6 (23.1)	0.045
Dyslipidemia, (%)	11 (64.7)	0 (0)	<0.001
Diabetes, n (%)	0 (0)	0 (0)	-
Arterial hypertension, n (%)	0 (0)	0 (0)	-
Plasma LDL cholesterol (mg/dL)	112.1 ± 33.6	89.3 ± 19.4	0.018
Plasma HDL cholesterol (mg/dL)	46.6 ± 11.0	62.9 ± 16.1	<0.001
Plasma triglycerides (mg/dL)	150.8 ± 74.1	82.4 ± 29.6	0.002
Triglyceride/HDL cholesterol ratio	3.551 ± 2.055	1.378 ± 0.596	<0.001
Serum ALT (U/L)	47.47 ± 25.54	23.06 ± 5.20	0.002
Serum AST (U/L)	33.33 ± 14.98	20.65 ± 4.33	0.010
Platelet count × 10^3^/µL	251.5 ± 58.5	249.0 ± 39.1	0.877
Leukocyte count × 10^3^/µL	5.54 ± 0.93	5.77 ± 1.29	0.960
Fasting LAL in whole blood (nmol/spot/h)	0.784 ± 0.239	1.086 ± 0.464	0.014
Carriers of the PNPLA3 I148M variant, n (%)	10 (62.5) *	13 (50.0)	0.530

* In one patient with MASLD, PNPLA3 rs738409 status was undetermined. Continuous variables are shown as mean ± SD. Categorical variables are shown as numbers and percentages. Abbreviations: LAL, lysosomal acid lipase; MASLD, metabolic dysfunction-associated steatotic liver disease.

**Table 2:** Pearson’s correlations between the difference in whole blood LAL activity four hours post-meal minus fasting blood LAL activity and plasma lipids in patients with MASLD and healthy liver controls.

	MASLD (N = 17)	CTRL (N = 26)
	LAL in Blood T4 Minus T0	LAL in Blood T4 Minus T0
LDL-C T0	0.431 (0.084)	**−0.491 (0.011)**
HDL-C T0	0.116 (0.659)	0.160 (0.435)
TG T0	0.182 (0.484)	−0.222 (0.276)
TG/HDL-C T0	0.091 (0.728)	−0.338 (0.092)
LDL-C T4	0.452 (0.069)	**−0.473 (0.015)**
HDL-C T4	0.201 (0.438)	0.180 (0.379)
TG T4	0.060 (0.818)	**−0.503 (0.009)**
TG/HDL-C T4	−0.043 (0.871)	**−0.512 (0.008)**
LDL-C T4 minus T0	−0.023 (0.931)	−0.111 (0.589)
HDL-C T4 minus T0	0.316 (0.216)	0.014 (0.945)
TG T4 minus T0	−0.052 (0.841)	**−0.502 (0.009)**
TG/HDL-C T4 minus T0	−0.115 (0.661)	**−0.505 (0.009)**

Numbers outside the parentheses indicate Pearson’s correlation coefficient r and numbers inside the parentheses indicate the two-tailed *p* value. Significant correlations are indicated in bold. Abbreviations: C, cholesterol; CTRL, control; LAL, lysosomal acid lipase; MASLD, metabolic-dysfunction-associated steatotic liver disease; TG, triglycerides.

**Table 3:** AUC T0T6 of plasma lipids in patients with MASLD and healthy liver controls in which plasma extracellular LAL and LPL activities were measured.

	MASLD (n = 8)	Controls (n = 9)	*p*
AUC LDL cholesterol	317.8 ± 84.2	201.5 ± 82.9	0.386
AUC HDL cholesterol	136.1 ± 29.8	193.6 ± 22.1	<0.001
AUC triglycerides	725.1 ± 313.7	398.8 ± 167.3	0.019
AUC triglyceride/HDL cholesterol ratio	17.912 ± 10.561	6.092 ± 2.233	0.010

Continuous variables are shown as mean ± SD. Categorical variables are shown as numbers and percentages. Abbreviations: AUC, area under the curve.

## Data Availability

The original contributions presented in this study are included in the article/Supplementary Materials, further inquiries can be directed to the corresponding author.

## Supplementary Materials

The following supporting information can be downloaded at: https://www.mdpi.com/article/10.3390/metabo14120725/s1, Supplementary Table S1: Macronutrient detail of the meal test. Supplementary Table S2. Pearson correlations between fasting or four-hour post-meal whole blood LAL activity and plasma lipids in MASLD patients and controls. Supplementary Table S3. Demographic and fasting metabolic characteristics of patients with MASLD and the controls without PNPLA3 rs738409 variant. Supplementary Table S4. Demographic and fasting metabolic characteristics of patients with MASLD and the controls with PNPLA3 rs738409 variant. Supplementary Table S5. Pearson correlations between the difference in whole blood LAL activity and plasma lipids in controls according to the presence or absence of the PNPLA3 rs738409 variant. Supplementary Table S6. Demographic, clinical, and fasting metabolic characteristics of subgroups of MASLD patients controls in which pre- and post-meal plasma extracellular LAL activity was measured. Supplementary Figure S1. Triglycerides-Cholesterol HDL ratio. Supplementary Figure S2. Scatter plots between the AUC T0T6 of the triglyceride/HDL-C ratio and those of the plasma enzymatic activities of LAL and LPL during a 6-h oral fat tolerance test. The AUC T0T6 triglyceride/HDL-C ratio vs. the AUC T0T6 LAL in MASLD patients (A) and in controls (B). The AUC T0T6 triglyceride/HDL-C ratio vs. the AUC T0T6 LPL in MASLD patients (C) and in controls (D) (Pearson correlations). AUC, area under the curve; LAL, lysosomal acid lipase; LPL, lipoprotein lipase; MASLD, metabolic dysfunction-associated steatotic liver disease. Supplementary Figure S3. Scatter plots between the fasting serum ALT concentration and the difference in extracellular LAL activity in plasma T4 minus T0. Fasting serum ALT concentration vs. difference in extracellular LAL activity in plasma T4 minus T0 in MASLD patients (A). Fasting serum ALT concentration vs. difference in extracellular LAL activity in plasma T4 minus T0 in controls (B).
