# Supplementary material for: Reduced Intra- and Extracellular Circulating Postprandial Lysosomal Acid Lipase Activity in Patients with MASLD
Source: Metabolites. 2024 Dec 23;14(12):725. doi: 10.3390/metabo14120725 (PMC11679640; doi:10.3390/metabo14120725)
Supplement: Supplementary file 1 [file metabolites-14-00725-s001.zip › Supplementary material 12-10-24.pdf]

Supplementary data to the manuscript entitled: "Reduced intra- and extracellular circulating postprandial lysosomal acid lipase response in patients with MASLD"

Monica Mischitelli[1], Eleonora Poggiogalle[2], Giulia Tozzi[3], Flaminia Ferri[1], Simona Parisse[1], Benedetta Meloni[1], Anna Morrone[1], Alice Sabbadini[1], Monther Salem[1], Elena Gangitano[2], Adriano De Santis[1], Giulia d'Amati[4], Lucio Gnessi[2], Lorenzo Maria Donini[2], Stefano Ginanni Corradini[1].

1. Department of Translational and Precision Medicine, Sapienza University of Rome
2. Department of Experimental Medicine, Sapienza University of Rome, Italy
3. Division of Metabolic Diseases, Bambino Gesù Children's Hospital IRCCS, Rome, Italy.
4. Department of Medical-Surgical Sciences and Biotechnologies Sapienza University of Rome, Rome, Italy

Table of contents:

1. Supplementary methods
2. Supplementary Table S1
3. Supplementary Table S2
4. Supplementary Table S3
5. Supplementary Table S4
6. Supplementary Table S5
7. References to supplementary methods
8. Figure legend to Supplementary Figure S1 attached as separate file
9. Figure legend to Supplementary Figure S2 attached as separate file
10. Figure legend to Supplementary Figure S3 attached as separate file

SUPPLEMENTARY METHODS

Study population

We recruited 43 participants to the study: 26 healthy controls without steatotic liver disease and 17 patients diagnosed with MASLD[1]. In all participants, liver ultrasound was performed by the same operator, who was blinded to the clinical and laboratory data of the participants. Study participants with overweight or obesity were consecutively enrolled among outpatients referred to the High Specialization Center for the Care of Obesity, Department of Experimental medicine, "Umberto I" University Hospital. Normal weight participants were recruited from hospital staff. The presence or absence of hepatic steatosis was evaluated ultrasonographically according to the criteria of Hamaguchi et al[2]. Nine patients with MASLD also underwent liver biopsy to verify the stage of fibrosis that may be present[3]. In the other patients with MASLD, advanced fibrosis was excluded using the Fibrosis-4 (FIB-4) score[4].

The inclusion criteria for all participants were age > 18 and < 70 years and being Italian of Caucasian ethnicity. Exclusion criteria were syndromic obesity, BMI > 35 kg/m^2, diabetes, arterial hypertension, history of neoplastic disease, any inflammatory or autoimmune disease, diagnosis of obstructive sleep apnea syndrome, corticosteroids for systemic use, any drug that may influence body weight or plasma lipids including statins, participation in a weight reduction program/exercise program in the last 3 months, renal failure (GFR<90 ml/min), heart failure (classes NYHA II-IV), history of chronic liver disease other than MASLD, advanced fibrosis or cirrhosis of any cause, excessive alcohol consumption (>140 g/week for men and 70 g/week for women), any therapy with antibiotics, bile salts, or cholestyramine within the last six months before enrollment, previous cholecystectomy, gallbladder disease. To further exclude subclinical liver disease in the controls, in addition to the absence of steatosis and complete normality of the liver ultrasound, subjects with serum ALT values greater than 30 U/L were excluded, even if our upper healthy ALT limit is < 45 U/L.

Oral fat tolerance test

Participants were asked to abstain from alcohol consumption, exercise, sugary drinks, changes in their usual eating pattern, and smoking in the 24 hours before the test meal. A standard high-fat meal was consumed as an oral fat tolerance test (OFTT) within 15 minutes. The OFTT was administered in the morning as breakfast, after a 12-hour fast. The fatty meal was provided by the Food Science and Human Nutrition Research Unit of the Department of Experimental Medicine and was represented by a muffin whose ingredients were olive oil, eggs, ricotta, nuts, cocoa and flour. The muffin was served with 150g of skimmed milk. Macronutrient details are shown in **Supplementary Table S1**. For all subjects, whole blood LAL activity was measured at fasting baseline (T0) and 4 hours after ingestion of the fatty meal (T4). In the last 17 participants (9 patients with MASLD and 8 controls), in whom LAL extracellular activity in plasma was also measured, postprandial blood samples were taken every 2 hours until the sixth hour after the end of the meal (T2, T4, T6). Blood samples were collected through a peripheral catheter placed in the cubital vein. After each blood draw, 2 ml of saline was infused into the intravenous catheter to prevent blood clotting in the catheter. Immediately before taking the next blood sample, the first ml of blood was discarded to avoid dilution of the blood sample. During the entire period up to the end of the postprandial blood sampling, no additional food or drink was permitted and participants remained inactive.

Analytical methods

LAL activity in whole blood was measured using 75 µl of blood in ethylene-diamine-tetra acetic acid (EDTA), with the dry blood spot (DBS) technique on filter paper (Whatman grade 903 Schleicher & Schuell). The blood was left to dry overnight at room temperature and then stored double-bagged with desiccant at -20°C. Within 1 week, the DBS sample was processed according to Hamilton et al.[5, 6].

Extracellular LAL activity in plasma was measured from blood in sodium citrate. Immediately after collection, 3 ml of blood was centrifuged at 1500 x g at 4°C for 10 minutes. Then 200 µl of

supernatant plasma was immediately carefully collected without disturbing the white buffy layer, frozen at -20 C and analyzed within 1 week.

The enzymatic assays were performed at the Bambino Gesù Hospital in Rome, Italy, by a biologist (G.T.) unaware of clinical, biochemical and genetic characteristics of any enrolled subject.

Forty µl of aqueous solution derived from 3.2 mm of DBS extracted in 200 µl of water, to measure LAL activity present in whole blood, or 40 µl of thawed plasma, to measure extracellular plasma LAL activity, were added to each reaction well and then processed as described by Hamilton et al. for LAL activity analysis from DBS samples[6]. Briefly, acid lipase activity was measured, in the presence of a buffer solution at pH 4.0 with 1.0% Triton X-100, with the fluorometric substrate 4-methylumbelliferyl palmitate and with cardiolipin as LAL activator. For each sample, one well was used for measuring the total acid lipase activity and one well for measuring the acid lipase activity in the presence of a specific LAL inhibitor (Lalistat 2 - Sigma-Aldrich, St. Louis, MO, USA), each in duplicate in a BioTek Synergy H1 Multimode Reader (Agilent). LAL activity was determined by subtracting the activity in the inhibited reaction from the uninhibited reaction and expressed as nmol/spot/h for whole blood and as nmol/ml/h for plasma. Inter- and intra-assay variations of LAL determinations were lower than 5%.

Plasma LPL enzymatic activity was measured with a fluorometric assay (Cell biolabs. Inc 7758 San Diego, CA). EDTA blood was centrifuged at 1000 x g at 4°C for 10 minutes. The plasma supernatant was collected without disturbing the buffy white layer and diluted from 1:50 to 1:200 according to the manufacturer's protocol of a fluorimetric assay (Cell biolabs. Inc 7758 San Diego, CA). The assay was performed using a fluorogenic triglyceride analogue as a lipase substrate at pH 8.0.

Measurements of triglycerides and LDL-C and HDL-C were performed using the colorimetric enzymatic reaction and those of serum aminotransferases using an enzymatic method with the Cobas® c 503 analytical unit, (Roche Diagnostics).

White blood cell and platelet counts were performed with the ADVIA® 2120i Hematology System (Siemens Healthineers).

Supplementary Table S1

Macronutrient detail of the meal test

**Macronutrients**	
Total fat (mainly long-chain fat)	72.6 g
Cholesterol	145.1 mg
Carbohydrate	51.8 g
Protein	22.4 g

Supplementary Table S2. Pearson correlations between fasting or four-hour post-meal whole blood LAL activity and plasma lipids in MASLD patients and healthy liver control subjects

	CTRLs			MASLD	
	LAL in blood T0	LAL in blood T4		LAL in blood T0	LAL in blood T4
LDL-C T0	0.314 (0.118)			-0.075 (0.774)	
HDL-C T0	0.152 (0.458)			0.194 (0.455)	
TG T0	-0.018 (0.929)			0.329 (0.197)	
TG/HDL-C T0	0.131 (0.523)			0.191 (0.462)	
LDL-C T4		-0-013 (0.948)			0.282 (0.273)
HDL-C T4		-0.148 (0.469)			0.332 (0.193)
TG T4		0.209 (0.307)			-0.083 (0.751)
TG/HDL-C T4		0.248 (0.222)			-0.229 (0.376)

Numbers outside the parentheses indicate the Pearson correlation coefficient r and numbers inside the parentheses indicate the two-tailed P value.

Abbreviations: C, cholestrol; TG, triglycerides

Supplementary Table S3. Demographic and fasting metabolic characteristics of patients with MASLD and the control group with healthy liver in subjects without PNPLA3 rs738409 variant.

	Men, n (%)	Age, years	BMI, kg/m^2	LDL-C (mg/dl)	HDL-C. (mg/dl)	TG (mg/dl)	TG / HDL-C ratio	LAL in blood (nmol/spot/hr)
MASLD-Wt (n=6)	4 (66.7)	56.0 ± 9.4	30.6 ± 2.9	128.0 ± 41.3	53.0 ± 12.1	165.7 ± 97.8	3.420 ± 2.500	0.847 ± 0.343
CTRL-Wt (n=13)	4 (30.8)	24.9 ± 2.6	22.5 ± 3.0	87.5 ± 16.3	62.2 ± 12.7	86.9 ± 30.8	1.451 ± 0.621	1.153 ± 0.522
P-Values	0.319	<0.001	<0.001	0.062	0.153	0.107	0.112	0.210

Continuous variables are shown as mean ± SD. Categorical variables are shown as numbers and percentages.

Abbreviations: BMI, body mass index; C, cholesterol; CTRL, control; LAL, lysosomal acid lipase; MASLD, Metabolic dysfunction-associated steatotic liver disease; TG, triglycerides; Wt, rs738409 PNPLA3 wild type

Supplementary Table S4. Demographic and fasting metabolic characteristics of patients with MASLD and the control group with healthy liver in subjects carrying the PNPLA3 rs738409 variant.

	Men, n (%)	Age, years	BMI, kg/m^2	LDL-C (mg/dl)	HDL-C. (mg/dl)	TG (mg/dl)	TG / HDL-C ratio	LAL in blood (nmol/spot/hr)
MASLD-I148M (n=10)	4 (40.0)	43.8 ± 11.7	29.7 ± 3.7	102.6 ± 28.5	45.4 ± 8.7	138.1 ± 68.1	3.382 ± 2.069	0.731 ± 0.161
CTRL-I148M (n=13)	5 (38.5)	26.0 ± 4.2	22.6 ± 3.1	91.1 ± 22.6	63.5 ± 19.5	77.8 ± 28.9	1.304 ± 0.585	1.018 ± 0.407
P-Values	1.000	<0.001	<0.001	0.163	0.004	0.009	0.005	0.018

Continuous variables are shown as mean ± SD. Categorical variables are shown as numbers and percentages.

Abbreviations: BMI, body mass index; C, cholesterol; CTRL, control; I148M, subjects with at least one mutated allele of the PNPLA3rs738409 variant; LAL, lysosomal acid lipase; MASLD, Metabolic dysfunction-associated steatotic liver disease; TG, triglycerides.

Supplementary Table S5. Pearson correlations between the difference in whole blood LAL activity four hours post-meal minus fasting LAL activity and plasma lipids in healthy liver controls according to the presence or absence of the PNPLA3 rs738409 variant

	**CTRL-Wt (n=13)**		**CTRL- I148M (n=13)**
	**LAL in blood T4 minus T0**		**LAL in blood T4 minus T0**
LDL-C T0	**-0.645 (0.017)**		-0.452 (0.121)
HDL-C T0	**0.559 (0.047)**		-0.178 (0.560)
TG T0	0.031 (0.919)		.0.529 (0.063)
TG/HDL-C T0	-0.320 (0.287)		-0.331 (0.270)
LDL-C T4	-0.479 (0.098)		-0.525 (0.065)
HDL-C T4	**0.661 (0.014)**		-0.216 (0.479)
TG T4	-0.540 (0.057)		-0.404 (0.170)
TG/HDL-C T4	**-0.589 (0.034)**		-0.272 (0.369)
LDL-C T4 minus T0	0.008 (0.978)		-0.220 (0.4699
HDL-C T4 minus T0	0.015 (0.961)		-0.103 (0.738)
TG T4 minus T0	**-589 (0.034)**		-0.257 (0.397)
TG/HDL-C T4 minus T0	**-0.598 (0.031)**		-0.195 (0.524)

Numbers outside the parentheses indicate the Pearson correlation coefficient r and numbers inside the parentheses indicate the two-tailed P value. Significant correlations are indicated in bold

Abbreviations: C, cholesterol; CTRL, control; I148M, rs738409 PNPLA3 variant carrier; LAL, lysosomal acid lipase; TG, triglycerides; Wt, rs738409 PNPLA3 wild type

Supplementary Table S6. Demographic, clinical, and fasting metabolic characteristics of subgroups of MASLD patients and healthy liver controls in which pre- and post-meal plasma extracellular LAL activity was measured

	**MASLD (n=8)**	**CONTROLS (n=9)**	**p**
Men, n (%)	3 (37.5)	6 (66.7)	0.347
Age, y	48.67 ± 9.42	26.25 ± 1.83	<0.001
Body Mass Index, kg/m^2	29.8 ± 4.0	22.4 ± 2.0	<0.001
LDL Cholesterol (mg/dl)	106.2 ± 26.5	90.4 ± 24.4	0.220
HDL Cholesterol (mg/dl)	44.3 ± 8.6	61.5 ± 8.6	0.002
Triglycerides (mg/dl)	173.8 ± 88.6	87.5 ± 42.2	0.024
Triglyceride/HDL cholesterol ratio	4.258 ± 2.376	1.404 ± 0.635	0.007
Serum ALT (U/L)	32.22 ± 15.51	20.38 ± 4.41	0.093
Serum AST (U/L)	50.22 ± 29.99	23.25 ± 4.20	<0.001
Platelet count × 10^3/µl	233.0 ± 58.9	273.4 ± 30.3	0.097
Leokocyte count × 10^3/µl	5.24 ± 0.95	5.89 ± 1.45	0.541
Fasting LAL in whole blood (nmol/spot/hr)	0.732 ± 0.263	0.924 ± 0.340	0.148
Fasting LAL in plasma (nmol/ml/hr)	8.55 ± 4.88	22.29 ± 11.11	0.004

Continuous variables are shown as mean ± SD. Categorical variables are shown as numbers and percentages.

Abbreviations: LAL, lysosomal acid lipase

References

1. Rinella ME, Lazarus JV, Ratziu V, Francque SM, Sanyal AJ, Kanwal F, et al. A multisociety Delphi consensus statement on new fatty liver disease nomenclature. Hepatology 2023; 78:1966-1986

2. Hamaguchi M, Kojima T, Itoh Y, Harano Y, Fujii K, Nakajima T, Kato T, et al. The severity of ultrasonographic findings in nonalcoholic fatty liver disease reflects the metabolic syndrome and visceral fat accumulation. Am J Gastroenterol. 2007;102:2708-2715.

3. Kleiner DE, Brunt EM, Van Natta M, Behling C, Contos MJ, Cummings OW, Ferrell LD, et al. Nonalcoholic Steatohepatitis Clinical Research Network. Design and validation of a histological scoring system for nonalcoholic fatty liver disease. Hepatology 2005; 41:1313-1321.

4. Srivastava A, Gailer R, Tanwar S, Trembling P, Parkes J, Rodger A, Suri D, et al. Prospective evaluation of a primary care referral pathway for patients with non-alcoholic fatty liver disease. J Hepatol. 2019; 71:371-378.

5. Ferri F, Mischitelli M, Tozzi G, Messina E, Mignini I, Mazzuca S, et al. Reduced Lysosomal Acid Lipase Activity in Blood and Platelets Is Associated With Nonalcoholic Fatty Liver Disease. Clin Transl Gastroenterol. 2020; 11:e00116.

6. Hamilton J, Jones I, Srivastava R, Galloway P. A new method for the measurement of lysosomal acid lipase in dried blood spots using the inhibitor Lalistat 2. Clin Chim Acta. 2012; 413:1207-1210.

Supplementary Figure S1: **Ratios of plasma lipid concentrations in MASLD patients compared to controls before and after 4 hours from the oral fat tolerance test.**

Triglyceride/HDL-C ratio at both T0 and T4 in MASLD and controls (A); AUC T0-T4 of the triglyceride/HDL-C ratio in MASLD and controls (B); difference between the triglyceride/HDL-C ratio at T4 minus that at T0 in MASLD and controls (C);

Data are reported as means and SD.

*p<0.05, ***p<0.001, ****p<0.0001 (Student's t test for independent or paired samples or Mann–Whitney U test or Wilcoxon test as appropriate).

AUC, area under the curve; LDL-C=LDL cholesterol, MASLD, metabolic dysfunction-associated steatotic liver disease; HDL=HDL cholesterol.

Supplementary Figure S2: **Scatter plots between the AUC T0T6 of the triglyceride/HDL-C ratio and those of the plasma enzymatic activities of LAL and LPL during a 6-hour oral fat tolerance test.** The AUC T0T6 triglyceride/HDL-C ratio vs the AUC T0T6 LAL in MASLD patients (A) and in controls (B). The AUC T0T6 triglyceride/HDL-C ratio vs the AUC T0T6 LPL in MASLD patients (C) and in controls (D) (Pearson correlations).

AUC, area under the curve; LAL, lysosomal acid lipase; LPL, lipoprotein lipase; MASLD, metabolic dysfunction-associated steatotic liver disease.

Supplementary Figure S3: **Scatter plots between the fasting serum ALT concentration and the difference in extracellular LAL activity in plasma T4 minus T0**.

Fasting serum ALT concentration vs difference in extracellular LAL activity in plasma T4 minus T0 in MASLD patients (A). Fasting serum ALT concentration vs difference in extracellular LAL activity in plasma T4 minus T0 in controls (B).
