# Supplementary figures and images for: Reduced Intra- and Extracellular Circulating Postprandial Lysosomal Acid Lipase Activity in Patients with MASLD
Source: Metabolites. 2024 Dec 23;14(12):725. doi: 10.3390/metabo14120725 (PMC11679640; doi:10.3390/metabo14120725)

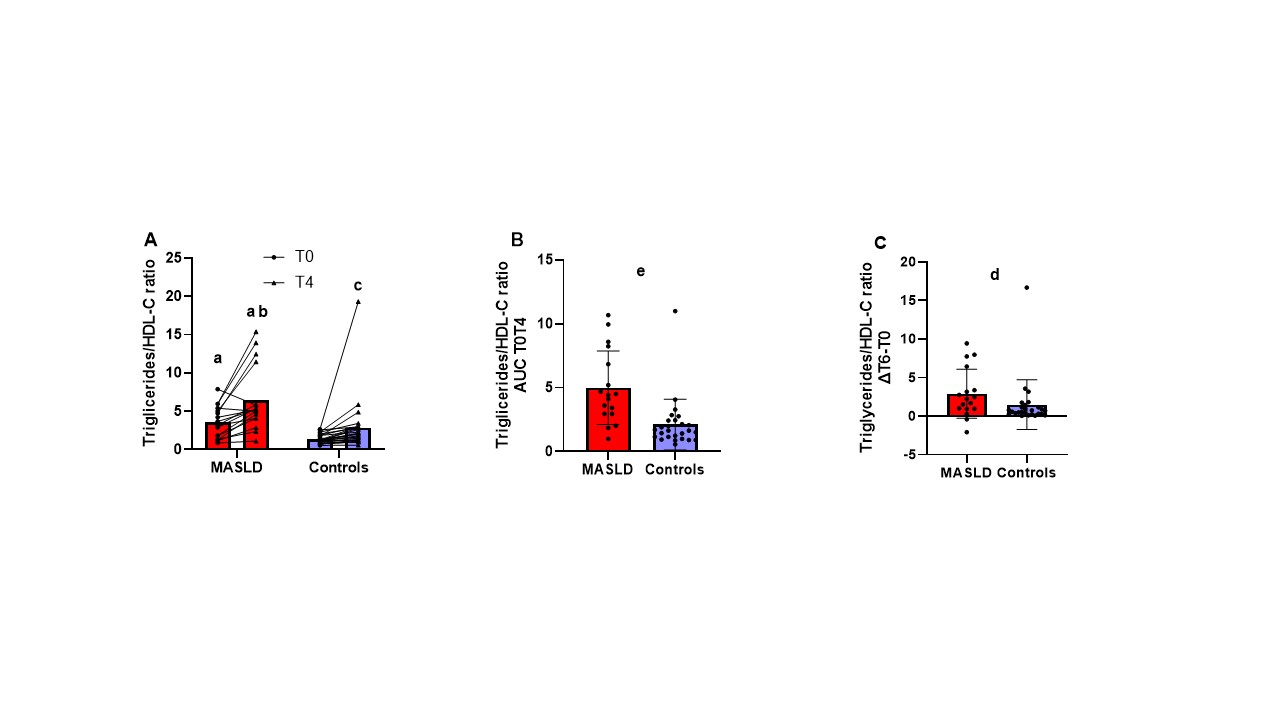

Supplement: Supplementary file 1 [file metabolites-14-00725-s001.zip › Supplementary Figure S1.jpg]

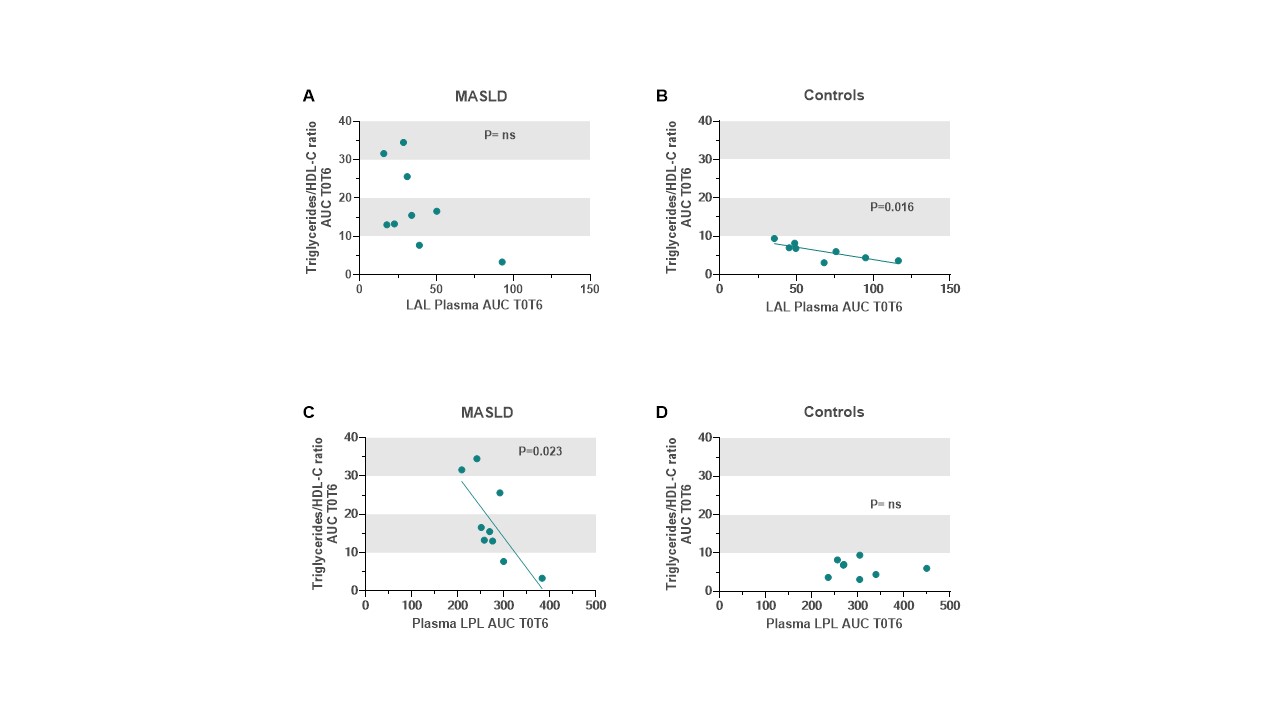

Supplement: Supplementary file 1 [file metabolites-14-00725-s001.zip › Supplementary Figure S2.jpg]

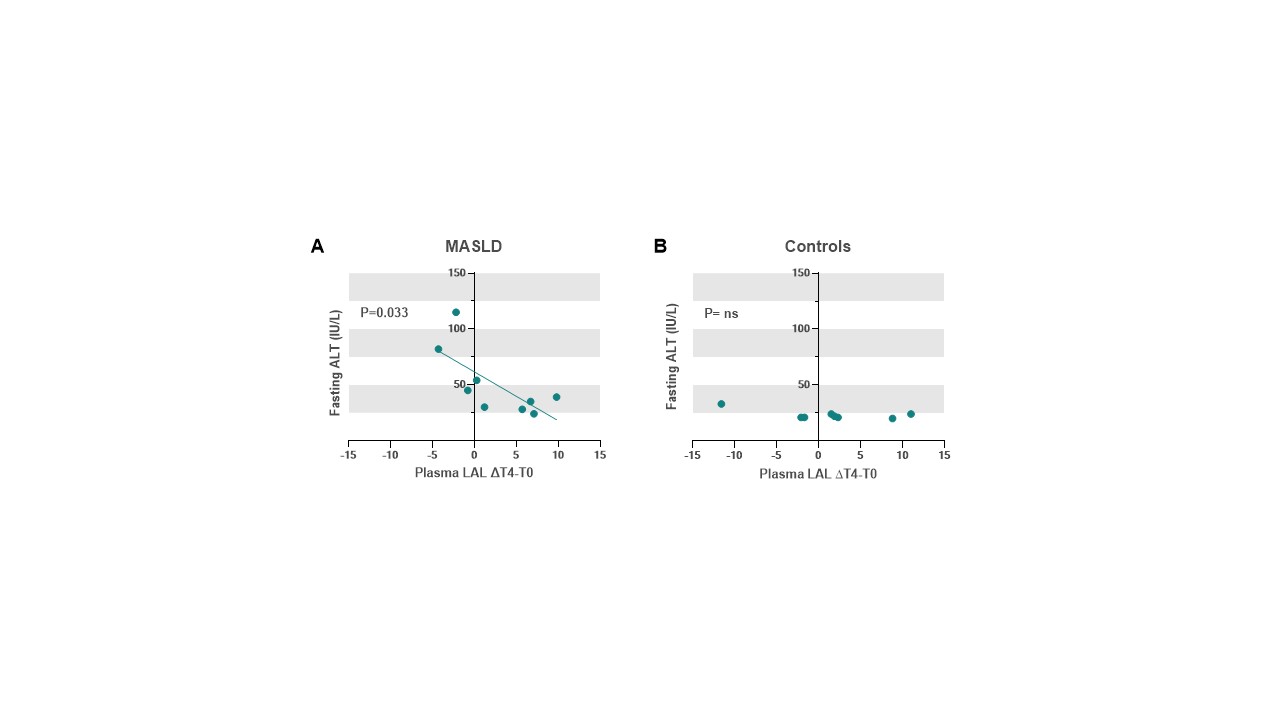

Supplement: Supplementary file 1 [file metabolites-14-00725-s001.zip › Supplementary Figure S3.jpg]
